# Chemical Constituents of Halophyte *Suaeda glauca* and Their Therapeutic Potential for Hair Loss

**DOI:** 10.3390/molecules29020298

**Published:** 2024-01-06

**Authors:** Yun-Na Kim, Min-Gyu Park, Yu-Jung Kim, Jae-Sun Lee, Bong-Oh Kwon, Jung-Rae Rho, Eun-Ju Jeong

**Affiliations:** 1 Department of Oceanography, Kunsan National University, Gunsan 54150, Republic of Korea; skdbssk@naver.com (Y.-N.K.); bongkwon@kunsan.ac.kr (B.-O.K.); 2 Department of Green Bio Science, Gyeongsang National University, Jinju 52725, Republic of Korea; qkralsrb8282@naver.com; 3 Agri-Food Bio Convergence Institute, Gyeongsang National University, Jinju 52725, Republic of Korea; aimeyj@naver.com (Y.-J.K.); 0869js@naver.com (J.-S.L.)

**Keywords:** *Suaeda glauca*, halophyte, compounds, dermal papilla cells, alopecia, hair loss

## Abstract

*Suaeda glauca*, a halophyte in the Amaranthaceae family, exhibits remarkable resilience to high salt and alkali stresses despite the absence of salt glands or vesicles in its leaves. While there is growing pharmacological interest in *S. glauca*, research on its secondary metabolites remains limited. In this study, chemical constituents of the aerial parts of *S. glauca* were identified using 1D- and 2D-NMR experiments, and its biological activity concerning hair loss was newly reported. Eight compounds, including alkaloids (**1**~**3**), flavonoids (**4**~**6**), and phenolics (**7** and **8**), were isolated. The compounds, except the flavonoids, were isolated for the first time from *S. glauca.* In the HPLC chromatogram, quercetin-3-*O*-β-d-glucoside, kaempferol-3-*O*-β-d-glucoside, and kaempferol were identified as major constituents in the extract of *S. glauca*. Additionally, the therapeutic potential of the extract of *S. glauca* and the isolated compounds **1**~**8** on the expressions of VEGF and IGF-1, as well as the regulation of Wnt/β-catenin signaling, were evaluated in human follicle dermal papilla cells (HFDPCs) and human umbilical vein endothelial cells (HUVECs). Among the eight compounds, compound 4 was the most potent in terms of increasing the expression of VEGF and IGF-1 and the regulation of Wnt/β-catenin. These findings suggest that *S. glauca* extract and its compounds are potential new candidates for preventing or treating hair loss.

## 1. Introduction

*Suaeda glauca* (Bunge) Bunge is a succulent halophyte belonging to the Amaranthaceae family, and is widely distributed throughout Asia. Beyond South Korea, this plant is also found in inland saline soil and salt marshes in China, Mongolia, Siberia, and Japan, highlighting its ecological significance and adaptability to various coastal and inland environments. This annual herb serves as both animal forage and a valuable resource in traditional Chinese medicine [1,2]. What sets *S. glauca* apart is its remarkable resilience to high salt and alkali stresses, thriving in environments with salt contents exceeding 0.48%, despite the absence of salt glands or vesicles in its leaves [3]. When subjected to salt stress, this resilient plant employs a sophisticated mechanism, accumulating organic acids and inorganic anions and thereby maintaining optimal internal ionic equilibrium by sequestering excess sodium ions into the vacuoles of its mesophyll cells [3,4]. In addition to its salt tolerance and medicinal qualities, *S. glauca* has been the subject of proteomics and metabolomics studies aimed at uncovering the mechanisms underlying its salt–alkali tolerance [5]. This research provides deeper insights into the plant’s adaptability to challenging environments. *S. glauca* is renowned for its potential in phytoremediation, particularly in saline soils, owing to its unique capability to hyperaccumulate sodium and its rapid biomass production [6,7].

Although the research on the secondary metabolites contained in *S. glauca* is limited, phenolic compounds, including flavonoids with hepatoprotective activity, have been reported in vitro [1,8]. In ultrasound-assisted extraction (UAE) for the enhanced extraction of gallic acid, known for its powerful antioxidant, anti-inflammatory, and anticancer properties, the content of gallic acid in *S. glauca* was found to range from approximately 0.62% to 0.89% [9]. Recently, the hepatoprotective effects of *S. glauca* were investigated in a CCl_4_-induced liver fibrosis animal model [10]. The administration of hot water extract of *S. glauca*, which was cultivated using a smart farming system with LED lamps orally for 6 weeks, reduced histological changes and collagen accumulation induced by CCl_4_ and demonstrated a decrease in hepatic stellate cell activation, inflammation, and an improvement in blood biochemical parameters. The anti-fibrotic effect of *S. glauca* extract was shown to be mediated through the inhibition of TGFβ1-Smad2/3 signaling in hepatic stellate cells.

The increasing prevalence of alopecia (hair loss), which induces psychological stress, has led to a growing interest in the development of hair loss treatments. The prevalence of male androgenetic alopecia varies depending on region and race, but it is known to increase with age, affecting approximately 30% to 50% of men by the age of 50 [11,12]. Dermal papilla cells and vascular endothelial cells have been utilized as excellent models for investigating the mechanisms involved in the prevention and treatment of hair loss, given their recognized significant roles in hair follicle formation, hair follicle morphogenesis, and the hair growth cycle.

Despite the increasing interest in the pharmacological activity of *S. glauca*, research on its secondary metabolites remains limited. In searching for candidates from halophytes native to Korea to treat or prevent alopecia, it was found that the methanolic extract of *S. glauca* effectively regulates the proteins related to alopecia in human follicle dermal papilla cells (HFDPCs) and human umbilical vein endothelial cells (HUVECs). In this study, we aimed to identify the bioactive compounds in *S. glauca* that may be effective in treating or preventing hair loss. The compounds were isolated from the aerial parts of *S. glauca* using chromatographic methods, and their chemical structures were elucidated through 1D and 2D-NMR experiments. Additionally, the chemical profile of the *S. glauca* extract was analyzed using HPLC. Furthermore, the regulatory effects of the extract and the isolated compounds on the expression of vascular endothelial growth factor (VEGF), insulin-like growth factor (IGF-1), β-catenin, and glycogen synthase kinase-3 beta (GSK-3β) were assessed in HFDPCs and HUVECs.

## 2. Results and Discussion

### 2.1. Isolation of Compounds ***1***~***8*** from Suaeda glauca

**Figure 1:**
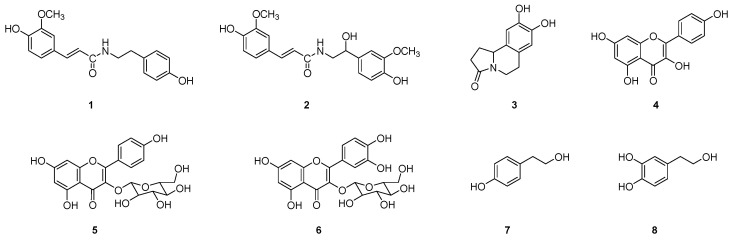
Chemical structures of **1**~**8** isolated from the aerial parts of *S. glauca*.

The aerial parts of *S. glauca* were extracted with an 80% aqueous solution of methanol. The ethyl-acetate-soluble fraction of *S. glauca* extract was subjected to repeated column chromatography and HPLC to obtain the eight (**1**~**8**) compounds. The structures of the isolated compounds were determined by 1D and 2D-NMR experiments. Compounds **1**~**8** were identified as N-trans-feruloyltyramine (**1**), N-feruloyl normetanephrine (**2**), trolline (**3**), kaempferol (**4**), kaempferol-3-*O*-β-d-glucoside (**5**), quercetin-3-*O*-β-d-glucoside (**6**), tyrosol (**7**), and hydroxy tyrosol (**8**) from their spectroscopic data by comparison with values reported in the literature (Figure 1) [13,14,15,16,17,18,19,20]. Compounds **1**~**3** belonged to alkaloids, **4**~**6** to flavonoids, and **7**~**8** to phenolics. All compounds, except for flavonoids (**4**~**6**), were reported for the first time from *S. glauca*. All compounds isolated herein from *S. glauca* are well known for exhibiting excellent antioxidant activity and health-improving effects. The antioxidant activity of flavonoids, including quercetin, kaempferol, and apigenin, is widely recognized. N-trans-feruloyltyramine, an alkaloid extracted from several plants, including *Polygonum sachalinensis*, *Tinospora tuberculata*, *Enicosanthum membranifolium*, etc., is also acknowledged as a potent antioxidant [21,22,23]. Tyrosol and hydroxytyrosol, along with their derivatives, are olive oil phenolic compounds. Their potential effects on human health, such as antiatherogenic, cardioprotective, anticancer, neuroprotective, and endocrine effects, have been previously reported [24].

### 2.2. HPLC Chemical Profile of Suaeda glauca Extract

**Figure 2:**
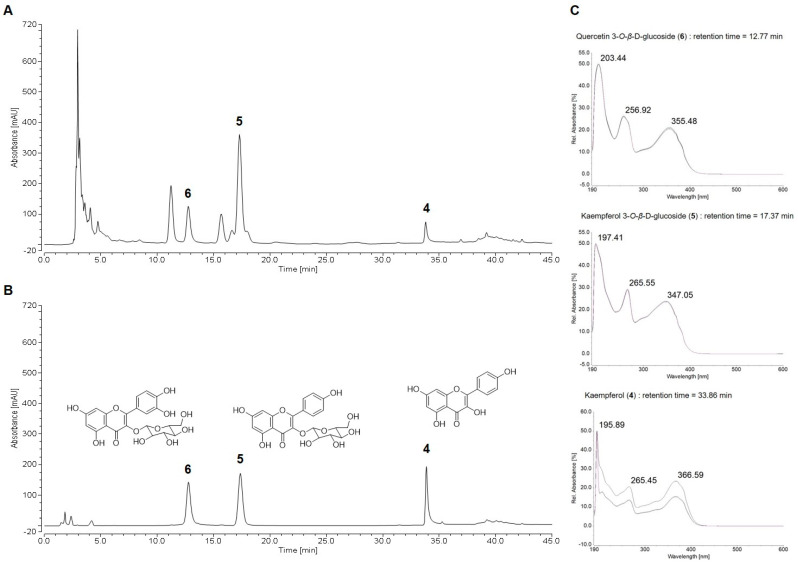
HPLC chromatograms of the extract of *S. glauca* (ESG) (**A**) and mixture of standard compounds: kaempferol (**4**), kaempferol-3-*O*-β-d-glucoside (**5**), quercetin-3-*O*-β-d-glucoside (**6**) (**B**), and UV spectra of each standard (**C**).

The qualitative analysis of the extract of *S. glauca* (ESG) was carried out on a reversed-phase C-18 column by HPLC. The ESG demonstrated five dominating signals in the HPLC chromatogram at UV 254 nm. The isolated compounds **1**~**8** were employed for chemical profiling of ESG. As a result, three major peaks out of five peaks were identified through the comparison of retention time and UV spectra of compounds **1**~**8**. As shown in Figure 2, quercetin-3-*O*-β-d-glucoside (**6**), kaempferol-3-*O*-β-d-glucoside (**5**), and kaempferol (**4**), with Rt values of 12.76, 17.30, and 33.80 min, were detectable at 254 nm.

### 2.3. The Effects of Suaeda glauca Extract on Wnt/β-Catenin Signaling and the Expressions of VEGF, IGF-1, p-GSK-3β in Human Follicle Dermal Papilla Cells (HFDPCs) and Human Umbilical Vein Endothelial Cells (HUVECs)

Dermal papilla cells are specialized intermediate layer cells located at the base of hair follicles. They function as reservoirs of multipotent stem cells, nutrients, and growth factors, playing a crucial role in hair follicle formation, hair follicle morphogenesis, and the hair growth cycle [25]. Recently, in three-dimensional tissue engineering for hair regenerative medicine, hair follicle germs containing vascular endothelial cells upregulated the expression of hair-morphogenesis-related genes in vitro. Additionally, there was a significant increase in the number of hairs regenerated after transplantation of hair follicle germs containing vascular endothelial cells into the dorsal skin of nude mice [26,27]. Due to the significant roles of dermal papilla cells and vascular endothelial cells in hair growth, these cells have been utilized as a model for investigating the mechanistic action of candidate substances on the cellular level of human hair follicles. Based on this, in the present study, we attempted to evaluate the therapeutic potentials of ESG and the isolated compounds on the expressions of proteins related to hair growth in HFDPCs and HUVECs. 

**Figure 3:**
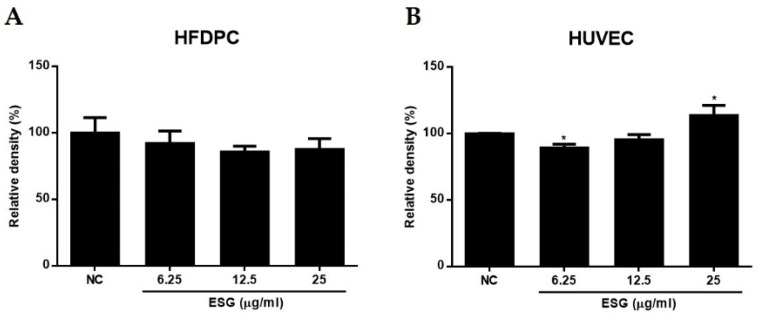
The effects of the extract of *S. glauca* (ESG) on cell viability of human follicle dermal papilla cells (HFDPCs) (**A**) and human umbilical vein endothelial cells (HUVECs) (**B**). Cells were treated with ESG (6.25, 12.5, and 25 μg/mL) for 24 h, and cell viability was determined by MTT assay. Results are presented as the mean ± S.D. of triplicate experiments; * *p* < 0.05 compared to non-treated control (NC).

Prior to evaluating the activities of ESG, the toxicities of ESG were measured using the 3-(4,5-Dimethylthiazol-2-yl)-2,5-Diphenyltetrazolium Bromide (MTT) assay. As shown in Figure 3, the treatment of ESG showed no toxicity against HFDPCs or HUVECs. At the concentration of 25 μg/mL of ESG, the cell viability of HUVECs was slightly increased.

**Figure 4:**
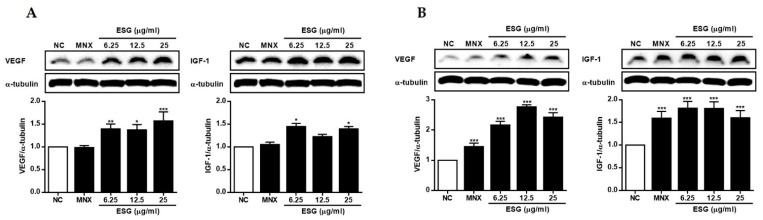
The effects of the extract of *S. glauca* (ESG) on the expressions of VEGF and IGF-1 in human follicle dermal papilla cells (HFDPCs) (**A**) and in human umbilical vein endothelial cells (HUVECs) (**B**). Cells were treated with ESG (6.25, 12.5 and 25 μg/mL) or MNX (10 μM, a positive control) for 24 h. The expression levels of each protein in cells were analyzed by Western blot. Results are presented as the mean ± S.D. of triplicate experiments; * *p* < 0.05, ** *p* < 0.01, *** *p* < 0.001 compared to NC. VEGF: vascular endothelial growth factor, IGF-1: insulin-like growth factor, MNX: minoxidil, NC: non-treated control.

**Figure 5:**
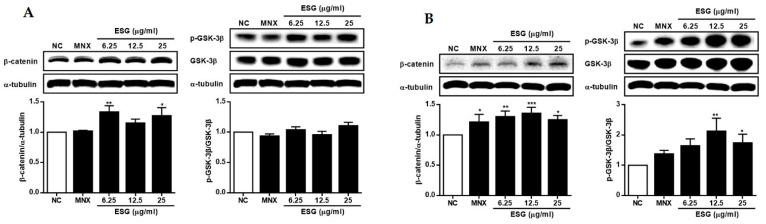
The effects of the extract of *S. glauca* (ESG) on the expressions of β-catenin and the phosphorylation of GSK-3β proteins in human follicle dermal papilla cells (HFDPCs) (**A**) and in human umbilical vein endothelial cells (HUVECs) (**B**). Cells were treated with ESG (6.25, 12.5, and 25 μg/mL) or MNX (10 μM, a positive control) for 24 h. The expression levels of each protein in cells were analyzed by western blot. Results are presented as the mean ± S.D. of triplicate experiments; * *p* < 0.05, ** *p* < 0.01, *** *p* < 0.001 compared to NC. GSK-3β: glycogen synthase kinase-3 beta, MNX: minoxidil, NC: non-treated control.

Based on MTT assay, the effects of ESG (25 μg/mL) on the expressions of VEGF, IGF-1, β-catenin, and GSK-3β were measured in HFDPCs and HUVECs (Figure 4 and Figure 5). In both in vitro systems, minoxidil was used as a positive control. Minoxidil is an FDA-approved medication for the treatment of hair loss. Alongside finasteride, it is widely used as a treatment for alopecia (hair loss). However, various attempts are underway to develop more effective and innovative therapeutic drugs due to the limited effectiveness and side effects of minoxidil.

The VEGF is known to be a molecule that specifically binds to endothelial cells, inducing endothelial cell proliferation, increased vascular permeability, and enhanced EC-mediated hyper-coagulability. Immunohistochemical evidence has indicated the virtual disappearance of VEGF from hair follicles in alopecia areata, and a similar trend has been observed in male pattern baldness. Thus, a reduction in or loss of VEGF production is believed to have a profound impact on maintaining proper vascular support for the skin, which could diminish the normal function of hair follicle cells [28]. To assess the regulatory effects of ESG on the expressions of VEGF and IGF-1, HFDPCs and HUVECs were treated with ESG (6.25, 12, and 25 μg/mL) (Figure 4). After 24 h of incubation, the expression levels of VEGF and IGF-1 proteins in cells were measured using Western blot analysis. It was observed that the expressions of VEGF and IGF-1 were increased by ESG in both cell lines. Treatment of HUVECs with ESG led to a significant increase in VEGF and IGF-1 expressions, comparable to the positive control, minoxidil, or even better.

The crucial role of the Wnt/β-catenin-mediated signaling pathway in governing hair follicle morphogenesis, hair shaft differentiation, and follicular recycling has been widely acknowledged [29,30,31,32,33]. Reddy et al. (2001) [34] demonstrated that specific Wnt ligands are selectively overexpressed at each stage of hair follicle differentiation, during which Wnt-5a has been shown to be expressed in a sonic hedgehog (SHH)-dependent manner. The stabilization of epidermal β-catenin leads to the formation of ectopic hair follicles and SHH expression [35]. Conversely, the absence of epidermal β-catenin results in the loss of SHH expression, suggesting that β-catenin plays a bridging role between Wnt signaling and SHH expression [36]. Reddy et al. (2001) [34] also illustrated that the removal of Wnt/β-catenin results in a reduction in the proliferation of hair follicle progenitor cells, whereas increasing Wnt/β-catenin signaling further enhances hair growth in mice [30]. Upon activation of Wnt signaling, GSK3β undergoes phosphorylation, leading to the inhibition of its activity. This results in elevated levels of β-catenin and its accumulation in the nucleus. In this nuclear environment, β-catenin serves as a transcriptional cofactor, facilitating the expression of cell growth factors by binding to Tcf/Lef transcription factors [37]. 

To determine whether ESG regulates Wnt/β-catenin signaling in HFDPCs and HUVECs, the expression level of β-catenin and the phosphorylation of GSK-3β in cells treated with ESG were measured. As shown in Figure 5, the expression of β-catenin protein was induced by ESG in both cell lines. While the phosphorylation of GSK-3β was not distinct in HFDPC cells, an increase in β-catenin expression and a concomitant rise in GSK-3β phosphorylation were observed in HUVEC cells. These results suggest that ESG promotes the phosphorylation of GSK-3β, resulting in an increased expression of the downstream signal β-catenin.

### 2.4. The Effects of ***1***~***8*** Isolated from S. glauca on Wnt/β-Catenin Signaling and the Expressions of VEGF, IGF-1, and p-GSK-3β in Human Follicle Dermal Papilla Cells (HFDPCs) and Human Umbilical Vein Endothelial Cells (HUVECs)

**Figure 6:**
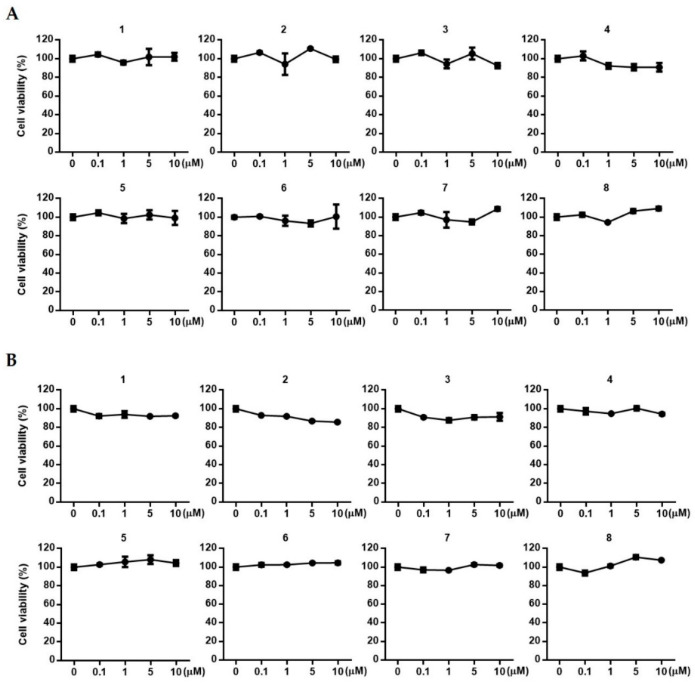
The toxicities of **1**~**8** isolated from *S. glauca* against human follicle dermal papilla cells (HFDPCs) (**A**) and human umbilical vein endothelial cells (HUVECs) (**B**). Cells were treated with each compound (0.1, 1.0, 5.0, and 10.0 μM) for 24 h, and cell viability was determined by MTT assay. Results are presented as the mean ± S.D. of triplicate experiments.

Based on the therapeutic potential of ESG to induce the expressions of VEGF and IGF-1, and to regulate Wnt/β-catenin in HFDPCs and HUVECs, we attempted to evaluate the activities of compounds **1**~**8** isolated from *S. glauca*. Prior to assays, the toxicities of **1**~**8** against HFDPCs and HUVECs were measured. As shown in Figure 6, no significant toxicity of the compounds was observed in either cell line in the concentration range from 0.1 to 10 μM.

**Figure 7:**
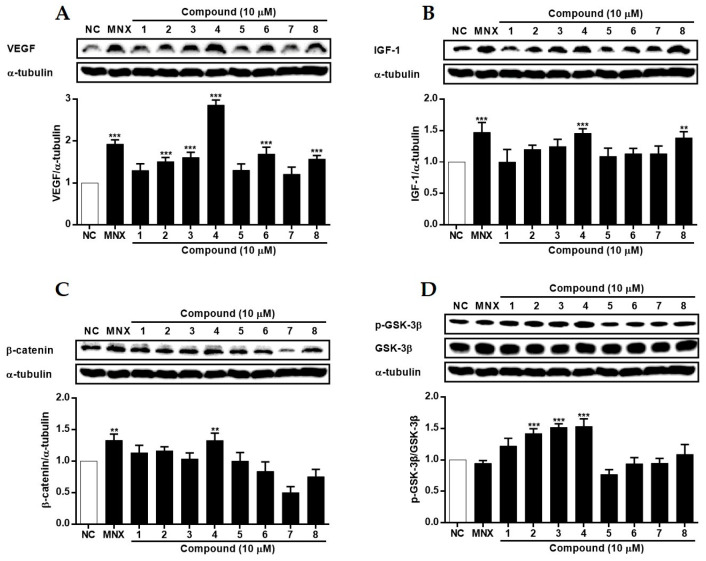
The effects of compounds **1**–**8**, isolated from *S. glauca*, on the expressions of VEGF (**A**), IGF-1 (**B**), β-catenin (**C**), and the phosphorylation of GSK-3β (**D**) proteins in human follicle dermal papilla cells (HFDPCs). Cells were treated with compounds (10 μM) or MNX (10 μM, a positive control) for 24 h. The expression levels of each protein in cells were analyzed by Western blot. Results are presented as the mean ± S.D. of triplicate experiments; ** *p* < 0.01 and *** *p* < 0.001 compared to NC. VEGF: vascular endothelial growth factor, IGF-1: insulin-like growth factor, GSK-3β: glycogen synthase kinase-3 beta, MNX: minoxidil, NC: non-treated control.

**Figure 8:**
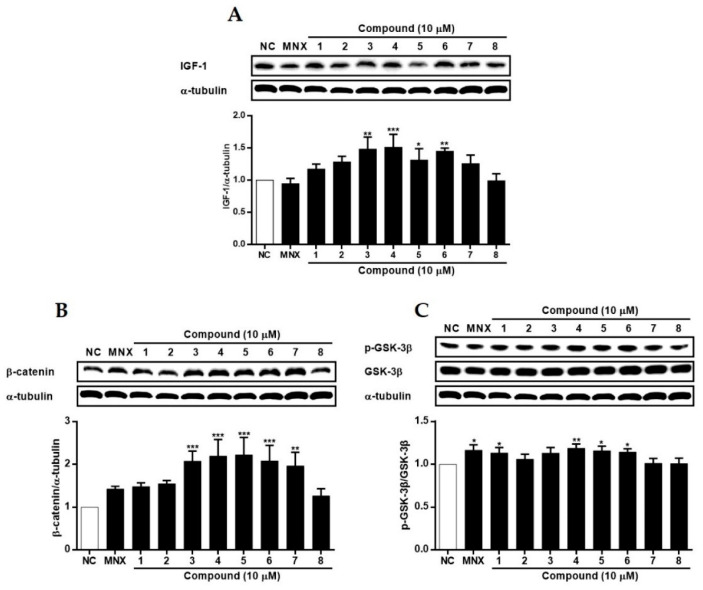
The effects of compounds **1**–**8** isolated from *S.glauca* on the expressions of IGF-1 (**A**), β-catenin (**B**) and the phosphorylation of GSK-3β (**C**) proteins in human umbilical vein endothelial cells (HUVECs). Cells were treated with compounds (10 μM) or MNX (10 μM, a positive control) for 24 h. The expression levels of each protein in cells were analyzed by Western blot. Results are presented as the mean ± S.D. of triplicate experiments; * *p* < 0.05, ** *p* < 0.01, *** *p* < 0.001 compared to NC. IGF-1: insulin-like growth factor, GSK-3β: glycogen synthase kinase-3 beta, MNX: minoxidil, NC: non-treated control.

In HFDPCs (Figure 7), the expression of VEGF was induced by all compounds except for **1**, **5**, and **7**, with a remarkable increase in cells treated with **4**. Additionally, the expression of IGF-1 increased with **4** and **8**. Concerning Wnt/β-catenin signaling, the expression of β-catenin was increased by **4**, and the phosphorylation level of GSK-3β showed a concomitant increase. The phosphorylation of GSK-3β was significantly induced by **2**, **3**, and **4**. In HUVECs (Figure 8), treatment of cells with **3**~**6** led to a significant increase in the expression of IGF-1. Also, the expression of β-catenin increased with **3**~**7**. Although the phosphorylation of GSK-3β was not significantly changed, a slight increase was observed in cells treated with **1** and **4**~**6**. While the regulatory activities of compounds on proteins related to hair loss (VEGF, IGF-1, β-catenin, and GSK-3β) varied between the two cell lines, compound **4** (kaempferol) exhibited the most superior activity. Recently, Lv et al. (2022) [38] reported molecular docking for targets related to alopecia areata. Kaempferol, along with quercetin and 7-methoxy-2-methyl isoflavone, demonstrated favorable docking results with targets IL-6, PTGS2, and TNF, indicating the potential of kaempferol to treat alopecia and related diseases.

## 3. Materials and Methods

### 3.1. Plant Material

*S. glauca* was collected from the foreshore in Sinan-gun, South Korea, in 2018. Plant identification was authenticated by Prof. Min Hye Yang, Pusan National University, Korea. A voucher specimen (voucher number GNT-69) was deposited at the Laboratory of Pharmacognosy, Gyeongsang National University, Korea.

### 3.2. Instrumentation and Reagents

High-resolution (HR) electrospray ionization (ESI) mass spectra were measured on a SCIEX X500R mass spectrometer (Sciex, Framingham, MA, USA). Nuclear magnetic resonance (NMR) spectra were recorded on a Varian VNMRS 500 NMR spectrometer (Varian, Palo Alto, CA, USA) operating at 500 MHz (^1^H) and 125 MHz (^13^C). All 1D and 2D NMR spectra were measured in methanol-*d*_4_ solution, which was referenced at 3.30 (^1^H) ppm and 49.0 (^13^C) ppm. Medium-pressure liquid chromatography (MPLC) was performed using Isolera (Biotage, Uppsala, Sweden) equipped with a UV detector. High-pressure liquid chromatography (HPLC) was performed using UltiMate 3000 (Dionex, Thermo Scientific, Karlsruhe, Germany) equipped with a pump (HPG-3200SD), autosampler (WPS-3000TSL analytical), temperature controller (TCC-3000SD), and diode array detector (DAD-3000). Open-column chromatography was performed with silica gel 60 (Merck Art: 9385, Darmstadt, Germany) and ODS gel (COSMOSIL 140 C18-OPN). Compounds were isolated and purified using HPLC with Thermo Acclaim™ Polar Advantage II C18 (5 μm, 4.6 × 250 mm), YMC PackPro C18 (5 μm, 10 × 250 mm), YMC J’Sphere ODS-H80 (4 μm, 10 × 250 mm), and Phenomenex synergi Polar (4 μm, 4.6 × 250 mm). The MTT reagent was purchased from Sigma-Aldrich (St. Louis, MO, USA). Methanol, *n*-hexane, CHCl_3_, EtOAc, and *n*-BuOH solvents were purchased from Daejung Chemical & Metals Co. Ltd. (Siheung-si, Republic of Korea). HPLC-grade methanol and water were purchased from Honeywell Burdick and Jackson (Muskegon, MI, USA).

### 3.3. Extraction and Isolation 

The freeze-dried aerial parts of *S. glauca* (2.5 kg) were ground and then extracted 3 times for 3 h with 80% (*v*/*v*) methanol using an ultrasonic apparatus. Removal of the solvent in vacuo was carried out, and 497.3 g of the crude extract was obtained. The dried methanolic extract of *S. glauca* was suspended in distilled water and sequentially partitioned based on polarity using *n*-hexane, CHCl_3_, EtOAc, and *n*-BuOH. The *n*-hexane, CHCl_3_, EtOAc, and *n*-BuOH soluble fractions were concentrated in vacuo and yielded 51.6 g, 6.9 g, 11.05 g, and 50 g, respectively. Then, the extract and each fraction were stored at −20 °C before use. 

The EtOAc fraction was subjected to silica gel column chromatography using mixtures of CHCl_3_-MeOH-water of gradient elution to yield twenty-three fractions (E1~23). The E20 fraction was subjected to MPLC (RP C18, MeOH-water, 5:95 → 70:30, 10.0 mL/min) to yield eighteen fractions (E20-1~18). Compounds **5** (4.5 mg) and **6** (6.7 mg) were isolated from the E20-8 fraction using HPLC (Thermo Acclaim™ Polar Advantage II C18, 5 μm, 4.6 × 250 mm, MeOH-Water 50:50, 1.0 mL/min). The E7 fraction was chromatographed via silica gel column eluting with a gradient of the mixture of CHCl_3_ and methanol to yield seventeen fractions (E7-1~20). The E7-7 fraction was further subjected to ODS gel chromatography (50% MeOH → 100% MeOH) to yield five fractions (E7-7-1~5). The E7-7-5 fraction was purified by HPLC (Thermo Acclaim™ Polar Advantage II C18, 5 μm, 4.6 × 250 mm, MeOH-Water 50:50, 1.0 mL/min) to isolate compound **4** (4.3 mg). The E7-9 fraction was applied to MPLC RP C18 gel chromatography with a gradient elution of water–methanol to yield fourteen fractions (E7-9-1~14). Compounds **7** (2.5 mg) and **8** (3.5 mg) were isolated from E7-9-1 fraction using HPLC (YMC PackPro C18, 4 μm, 4.6 × 250 mm, MeOH-Water, 20:80, 1.5 mL/min). Compound **1** (0.9 mg) was isolated from the E7-9-6 fraction using HPLC (YMC J’Sphere ODS-H80, 4 μm, 10 × 250 mm, MeOH-Water, 45:55, 1.5 mL/min). Compound **2** (1.5 mg) was isolated from the E7-9-4 fraction using HPLC (Phenomenex synergi Polar, 4 µm, 4.6 × 250 mm, MeOH-water 40:60, 1.0 mL/min). Compound **3** (3.0 mg) was isolated from the E7-9-2 fraction using HPLC (Phenomenex synergi Polar, 4µm, 4.6 × 250 mm, MeOH-Water 25:75, 1.0 mL/min). 

### 3.4. HPLC Chromatographic Conditions

The methanolic extract and the mixture of isolated compounds were dissolved in 50% methanol and methanol, respectively. Then, each dissolution was filtered through a 0.45 μm PVDF membrane filter (Hyundai Micro, Seoul, Republic of Korea, PVDF, 25 mm) and analyzed using HPLC.

Chromatographic separation of the methanolic extract of *S. glauca* and the mixture of isolated compounds was achieved with a Phenomenex synergi 4 μm Polar (4.6 × 250 mm) using elution with water (A) and methanol (B) as a mobile phase under the following gradient conditions: 0–25 min, linear from 45% to 50% B; 25–35 min linear from 50% to 80% B; 35.1–45 min, 95% B. The flow rate was 1.0 mL/min at 25 °C, and the eluent was detected with DAD at 254 nm.

### 3.5. Cell Cultures

HFDPCs were purchased from PromoCell (Heidelberg, Germany) and were cultured in a follicle dermal papilla cell growth medium kit (Promocell, Heidelberg, Germany) supplemented with 1% (*v*/*v*) penicillin/streptomycin (P4333, Sigma-Aldrich, St. Louis, MO, USA). HFDPCs were used within four passages. HUVECs were purchased from Thermo Fisher Scientific (USA) and were cultured in endothelial cell basal medium-2 (EBM-2, Lonza, Walkersville, MD, USA) supplemented with EGM™-2 SingleQuots^®^ supplement (Lonza, Walkersville, MD, USA). The cells were incubated at 37 °C and 5% CO_2_ saturation.

### 3.6. Estimation of Cell Viability

Cell viability was determined using the colorimetric MTT assay, which is based on the reduction of MTT to formazan by cellular dehydrogenase. HFDPCs and HUVECs were seeded at densities of 1 × 10^4^ cells/well, respectively, in 96-well plates and incubated for 24 h. Then, they were treated with a test sample and cultivated for 24 h. MTT (2 mg/mL) in distilled water was added to each well for 3 h at 37 °C. The supernatant was then aspirated and 100 µL of DMSO was added to dissolve the formazan. After insoluble crystals were completely dissolved, absorbance at 540 nm was measured using a microplate reader. Data were expressed as percentage cell viability relative to control cultures.

### 3.7. Western Blotting

HFDPCs and HUVECs were seeded at a density of 3 × 10⁵ cells/mL in six-well plates and incubated overnight. The cells were treated with minoxidil (10 μM); 6.25, 12.5, and 25 μg/mL of ESG extracts; and 10 μM compound for a further 24 h. The cells were washed with cold phosphate-buffered saline (PBS), and cell lysates were extracted with a lysis buffer (M-PER Mammalian Protein Extraction Reagent 78501, Thermo Scientific, Waltham, MA, USA) containing a protease inhibitor cocktail (Thermo Scientific, Waltham, MA, USA). The protein extracts were centrifuged at 13,000 rpm for 20 min at room temperature. The protein content of the cell lysate was quantified using the Bradford assay. Thirty grams of harvested proteins were separated using 10% SDS-PAGE at 100 V and transferred to polyvinylidene fluoride (PVDF) membranes. The membranes were blocked with 5% skim milk for 1 h at room temperature. The membranes were incubated with 1:1,000 diluted primary antibodies GSK-3β, p-GSK-3β (Cell Signaling Technology, Inc., Danvers, MA, USA), VEGF, IGF-1, β-catenin, and α-tubulin (Santa Cruz Biotechnology, Santa Cruz, CA, USA) at 4 °C overnight. After washing three times with TBST, the immunoreactive bands were visualized by using immunopure peroxidase-conjugated mouse anti-rabbit IgG and goat anti-mouse IgG (1:10,000 dilution; Santa Cruz Biotechnology, Santa Cruz, CA, USA). Membranes were incubated with secondary antibodies for 1 h at room temperature. Blots were washed three times with TBST buffer. Protein bands were visualized using ECL solution (Bio-Rad, Hercules, CA, USA) and calibrated using the Chemidoc Imaging System (Fusion FX5, Vilber Lourmat, Collégien, France). The density value of the protein bands was normalized to α-tubulin.

## 4. Conclusions

With the growing demand for new materials for treating or preventing hair loss, we attempted to identify potent candidates from halophytes that regulate enzymes related to hair loss. In a screening experiment utilizing HFDPCs and HUVECs as an in vitro system, the methanol extract of *S. glauca* exhibited excellent activity. From the ethyl-acetate-soluble fraction of *S. glauca* extract, eight compounds, including three alkaloids, three flavonoids, and two phenolics, were successfully isolated, and the structures of the isolated compounds were identified through NMR experiments. In an HPLC chromatogram of ESG, five dominating signals were detected at UV 254 nm, with three major peaks identified as quercetin-3-*O*-β-d-glucoside, kaempferol-3-*O*-β-d-glucoside, and kaempferol. In HFDPCs and HUVECs, the pre-treatment of ESG regulated Wnt/β-catenin signaling and increased the expressions of the VEGF and IGF-1 proteins. Among the eight compounds isolated from *S. glauca*, compound **4** (kaempferol), one of the main constituents detected in the HPLC chromatogram of ESG, exhibited the most potent activity. The treatment of HFDPCs or HUVECs with **4** led to a significant increase in the expressions of VEGF, IGF-1, and β-catenin, as well as the phosphorylation of GSK-3β, which was comparable to the positive control, minoxidil, or even better. These results suggest that the halophyte *S. glauca*, containing bioactive flavonoids, is a useful therapeutic agent for the treatment of alopecia and related diseases.

## Data Availability

Data are contained within the article.
